# Plant Polysaccharides Modulate Immune Function via the Gut Microbiome and May Have Potential in COVID-19 Therapy

**DOI:** 10.3390/molecules27092773

**Published:** 2022-04-26

**Authors:** Mengsheng Tang, Lu Cheng, Yanan Liu, Zufang Wu, Xin Zhang, Songmei Luo

**Affiliations:** 1 Department of Food Science and Engineering, Ningbo University, Ningbo 315211, China; tms18715016356@163.com (M.T.); wzfwpf@163.com (Z.W.); zhangxin@nbu.edu.cn (X.Z.); 2 Department of Food Science, Rutgers University, New Brunswick, NJ 08901, USA; lc894@scarletmail.rutgers.edu; 3 Department of Pharmacy, Lishui Central Hospital, Lishui 323000, China

**Keywords:** COVID-19, plant polysaccharides, probiotics, SCFAs, immunity, gut flora

## Abstract

Plant polysaccharides can increase the number and variety of beneficial bacteria in the gut and produce a variety of active substances, including short-chain fatty acids (SCFAs). Gut microbes and their specific metabolites have the effects of promoting anti-inflammatory activity, enhancing the intestinal barrier, and activating and regulating immune cells, which are beneficial for improving immunity. A strong immune system reduces inflammation caused by external viruses and other pathogens. Coronavirus disease 2019 (COVID-19) is still spreading globally, and patients with COVID-19 often have intestinal disease and weakened immune systems. This article mainly evaluates how polysaccharides in plants can improve the immune system barrier by improving the intestinal microecological balance, which may have potential in the prevention and treatment of COVID-19.

## 1. Introduction

COVID-19 is caused by human infection with the SARS-CoV-2 virus. It is a coronavirus that directly infects the human lower respiratory tract and causes severe pneumonia. Its clinical manifestations are the same as those of severe acute respiratory syndrome and Middle East respiratory syndrome (MERS) [1]. Most patients with COVID-19 develop fever, cough, weakness and fatigue after diagnosis [2]. A considerable number of patients may have physical pain, uncontrollable runny nose, loss of taste and smell, sore throat and diarrhea caused by upper respiratory tract infection [3]. Respiratory viral infections lead to disturbances in the gut microbiota, while beneficial microbes tend to have less space in immunocompromised populations [4]. It is hypothesized that there is also a reduction in beneficial bacteria in COVID-19 patients’ guts. Senior citizens and those with underlying health problems may have more serious symptoms. The World Health Organization (WHO) has pointed out that COVID-19 has become a global pandemic trend because of its severity and global spread. The spread of the virus from person to person through respiratory droplets or direct contact accelerates the infection rate [5]. In this short period, the epidemic has infected millions of people in more than 200 countries [6].

Human gut flora and its metabolites play important roles in both local mucosal immunity and systemic immunity. Intestinal microorganisms have the potential to induce local and systemic inflammation [7]. The intestinal mucosal physical barrier and immune barrier can protect the host from microbial infection, limit the damage of inflammatory tissue and produce an adaptive immune response [8]. Once these immune responses are unbalanced, microbial invasion of the intestine will lead to idiopathic inflammatory diseases. In the case of low immunity, a considerable number of inflammatory leukocytes, such as neutrophils, monocytes and lymphocytes, will enter the intestinal lamina propria, directly or indirectly leading to tissue damage or dysfunction [9]. Therefore, the human body will suffer from edema, goblet cell destruction, fibrosis, erosion and ulcer. Once human immunity drops, the body will be more vulnerable to the invasion of external, potentially unsafe factors. The international consensus definition of probiotics is “live microorganisms that, when administered in adequate amounts, confer a health benefit to the host” [10]. The ability of probiotics and their metabolites to downregulate inflammatory mediators and increase epithelial barrier function may be the most important in the inflamed gut [11], while the ability to increase colonic short-chain fatty acids (SCFAs) and hydration contributes more to normal gut motility [12]. Probiotics have been shown to increase phagocytosis or the activity of natural killer cells [13]. upregulate antibody secretion and increase anti-inflammatory factors [14]. A variety of actions have beneficial effects on the human immune system.

Most of the prebiotics that have been found are sugars, such as polysaccharides and starches. However, other substances, such as polyphenols and polyunsaturated fatty acids, can also be classified as prebiotics due to their beneficial effects on the host by modulating the gut flora [15]. Polysaccharides are active polymer materials composed of monosaccharides connected by glycoside chains. In recent years, polysaccharides have become the focus of immunopharmacology for immune regulation, antiradiation and antiviral activities [16]. Previous studies also suggested that polysaccharides can effectively regulate intestinal flora, which is mainly achieved by increasing the number and type of probiotics in the intestine and the metabolites of probiotics [17]. Although the pharmacological mechanisms of polysaccharides are not entirely revealed, researchers have reached a consensus on the probiotic effects of polysaccharides. Therefore, exploring the effects of plant polysaccharides on the immune system by modulating the intestinal flora has become a hot spot. This review aims to show how host immunity can be enhanced through intestinal flora by plant polysaccharides and their metabolites, which may provide new ideas for the prevention or treatment of COVID-19.

## 2. Polysaccharides Ameliorate COVID-19

**Figure 1:**
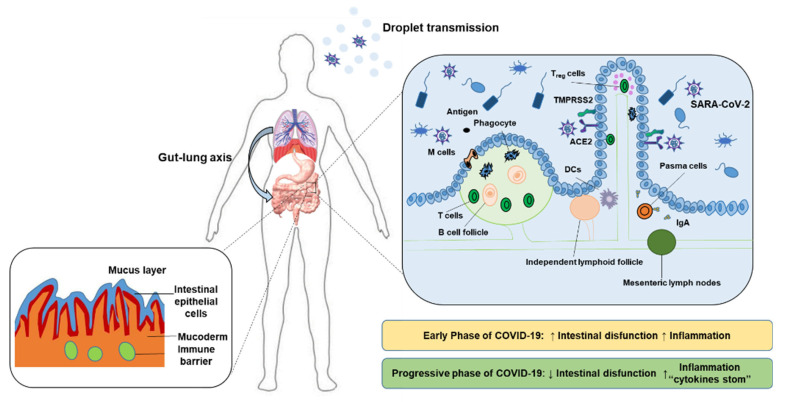
The important immune barrier of human intestine and the mechanism of COVID-19 invading human body.

COVID-19 is caused by human infection with the SARS-CoV-2 virus, and it belongs to *Coronaviridae*. Viral spikes were observed around the virus under an electron microscope. Once the human body is infected, the virus will attack the host’s respiratory system, cardiovascular system, central nervous system and gastrointestinal system [18]. Proximity to patients during dental care, the generation of large amounts of aerosols and the identification of SARS-CoV-2 in saliva suggest that the oral cavity is a potential reservoir for COVID-19 transmission [19]. Shortly after the outbreak of the epidemic, the study found two key molecules of viral invasion: the ACE2 receptor and TMPRSS2. The ACE2 receptor is the target of viral invasion into the human body. ACE2 is thought to be widely expressed in the heart, kidney, brain, lung and digestive tract. The spike protein of SARS-CoV-2 binds to the ACE2 receptor. Compared with SARS-CoV S protein, SARS-CoV-2 has a higher affinity for ACE2 receptors in the human body, which reveals why COVID-19 has such strong transmission ability. TMPRSS is a protease on the cell surface. It activates the spike protein on the virus surface, promotes the combination of the spike protein and ACE2 in host cells and causes a series of clinical manifestations [20] (Figure 1). People with low immunity, such as the elderly and infants, may develop acute respiratory distress syndrome (ARDS), cardiovascular disease and other serious complications [21].

In the analysis of clinical symptoms of patients with COVID-19, it was pointed out that some patients had observed mild initial gastrointestinal symptoms before fever and dry cough. SARS-CoV-2 RNA has been found in patients’ respiratory secretions and feces. Combined with the clinical symptoms of abdominal pain and diarrhea in other patients, it can be concluded that the gut–lung axis and intestinal flora are involved in the regulation of COVID-19 [22]. Existing research evidence defines the gut–lung axis as follows: “intestinal microorganisms affect human lung health through an important cross dialogue between intestinal flora and lungs.” Obviously, this is a two-way mechanism similar to the gut–brain axis. Through this mechanism, endotoxin and intestinal microbial secretions affect lung function via the blood. When lung inflammation occurs, it will lead to intestinal microecological imbalance. In the intestines of patients with COVID-19, few *Bifidobacteria* with immunomodulatory ability were found. It has been confirmed that the number of T cells, CD4 + T cells and CD8 + T lymph nodes in patients decreased sharply [23]. At the same time, the levels of some inflammatory cytokines and chemokines in patients are consistent with those in patients with severe diseases.

Although we have not yet given a definite plan for the treatment of COVID-19, we can imagine that the regulatory mechanism of polysaccharides and the effects of their metabolite SCFAs on intestinal flora enhance immunity or establish a strong defense system for the body and will alleviate the clinical symptoms of inflammation caused by SARS-CoV-2 and intestinal flora disturbance. Food rich in polysaccharides has immune regulation characteristics and is the most valuable regulator for immune response. Therefore, the use of polysaccharides obtained from food provides an innovative strategy to prevent the serious side effects of viral infection [24]. In one study [25], four marine sulfated polysaccharides were screened for their inhibitory activity against SARS-CoV-2, including sea cucumber sulfated polysaccharide (SCSP), fucoidan from brown algae, iota-carrageenan from red algae and chondroitin sulfate C from shark (CS). Furthermore, a test using a pseudotype virus with S glycoprotein confirmed that SCSP could bind to the S glycoprotein to prevent SARS-CoV-2 host cell entry. These four polysaccharides are promising inhibitors of SARS-CoV-2 infection. These crude polysaccharides have strong antiviral activity [26]. The chemical composition of polysaccharides in natural foods, such as mushrooms, yeast, fruits, algae and grains, can produce these biopolymers, which act as powerful substances to purify the activated immune system, especially the adaptive system [27]. Furthermore, in addition to antioxidant activity, these biopolymers also have interesting biological properties [28], anti-inflammatory [29] and antiviral [30] activities, biocompatibility, biodegradability and non-toxicity [31]. They can be used in innovative therapies. In sum, there is a precedent for the treatment of SARS virus infection. Therefore, polysaccharides can be used as an activator of the immune system to reduce the damage caused by infectious factors such as SARS-CoV-2.

## 3. Mechanism of Polysaccharides Regulating Gut Flora

Experts explain prebiotics as follows: some organic substances cannot be digested and absorbed by the host but selectively promote the metabolism and proliferation of beneficial bacteria in the intestine so as to improve the health of the host [32]. Based on this concept, it can be recognized that plant polysaccharides belong to one of the prebiotics beneficial to host health [33]. Prebiotics such as plant polysaccharides can be inhaled into the body as a carbon source of intestinal flora, providing conditions for the proliferation of beneficial bacteria and the growth and development of intestinal immune organs [34]. Plant polysaccharides have a variety of activities, and they have become a research hot spot in many fields such as medicine and food due to their safety, high efficiency and low toxicity. Plant polysaccharides have various biological activities, such as anti-liver injury, antitumor, antivirus, hypolipidemic, anticoagulation, antioxidation and immune regulation properties. Its chemical structure is the basis of its biological activity [35]. More and more scientific studies show that the health of intestinal flora is inseparable from the physiological activity of plant polysaccharides [36].

### 3.1. Increase the Number and Types of Probiotics in Intestinal Microecology

*Dendrobium* polysaccharides modulate gut microbiota composition and metabolism by enhancing *Romboutsia*, *Lactobacillus* and *Odoribacter* and reducing *Parasutterella*, *Burkholderia-Caballeronia-Paraburkholderia* and *Acinetobacter* in mice with colitis. This indicates that the gut microbiota is involved in restoring the stability of the gut microbiome under inflammatory conditions. Notably, *Dendrobium* polysaccharides significantly restored Th17/Treg cell homeostasis and the expression of specific cytokines. Western blotting of colon tissue also showed that Dendrobium polysaccharide significantly upregulated the expression of Nrf2 and inhibited the phosphorylation of NF-kB signaling [37]. The aim of another study was to investigate the effect of prebiotic intake (including oligofructose, oligosaccharides, polyglucose and resistant dextrin) on immune function and gut microbiome structure in perioperative patients with colorectal cancer (CRC). Experimental nodes showed that taking prebiotics 7 days before surgery improved serum immune measures in patients with colorectal cancer. Meanwhile, prebiotics improved the abundance of four symbiotic microbiomes containing conditionally pathogenic bacteria in patients with colorectal cancer [38]. Observational studies showed that eating whole grains (WG) was negatively associated with inflammation. However, the evidence from interventional studies is limited, and few studies have included measurements of cell-mediated immunity. Thus, the researchers assessed a diet rich in WGs and refined grains (RGs) for immune and inflammatory responses in healthy adults, gut microbiota and microbial products. The conclusion was that short-term intake of WGs in a weight-maintenance diet increases stool weight and frequency, has some positive effects on the gut microbiota, SCFAs, effector memory T cells and the acute innate immune response, and shows no influence on other cell-mediated immune or systemic and intestinal inflammation markers [39]. In sum, the intestinal flora can regulate the body’s immunity, and a change in the intestinal microbiome is bound to cause the enhancement or weakening of the body’s immune function.

Intestinal microorganisms participate in mucosal immunity, in which probiotics stimulate the mucosal immune system and induce signal networks mediated by the whole bacterium or its cell wall structure [40]. Probiotics interact with various cell types through cytokines, such as enterocytes, dendritic cells (DCs), and Th1 (helper T cells), Th2 and Treg cells (regulatory T cells), thereby regulating the immune system or promoting anti-inflammatory effects. To date, the beneficial effects of oral or nasal probiotics have been demonstrated in two mouse models of infection (influenza and pneumonia). Their protective effect is mediated by specific immune modulation and is distinguished by the early recruitment of innate leukocytes in the lung, displaying potent killing properties [41]. These effects help to reduce intestinal permeability, thereby reducing endotoxin and systemic inflammation. The gut microbiota participates in the regulation of human psychiatric diseases such as depression through the “gut-brain axis” and relieves intestinal inflammation in patients with depression [42]. In a study using gluten-free or gluten-containing diets to evaluate the effects of probiotic supplementation on mental state, inflammation and intestinal barrier in patients with major depressive disorder (MDD), the results obtained showed that the combination of a gluten-free diet and probiotic supplementation may inhibit the immune–inflammatory cascade in the process of MDD and improve the characteristics of mental health and the intestinal barrier [43]. To some extent, probiotic supplementation decreases the body’s inflammation.

### 3.2. An Important Metabolite of Gut Flora—Short-Chain Fatty Acids

Plant polysaccharides use the beneficial flora in the intestinal flora as a medium, repair the intestinal barrier and enhance intestinal resistance through a variety of ways: increasing the expression of tight junction proteins and repairing the damaged intestinal barrier [44]. By increasing the expression of tight junction proteins and promoting the formation of the intestinal epithelial barrier, bacterial translocation, intestinal inflammation and metabolic endotoxemia were significantly reduced [45]. Indigestible carbohydrates such as dietary fiber, resistant starch and oligosaccharides in the colon are metabolized by beneficial bacteria such as *Lactobacillus* and *Bifidobacterium* to produce SCFAs. Animal experiments have proved that in the rat model, intravenous injection of a certain amount of SCFAs maintains the height, width, recess depth and mucosal thickness of small intestinal villi at a healthy standard. It enhances the function of the epithelial barrier by increasing the production of mucin and strengthening the connection between mucin and goblet cells [46]. This is important for intestinal barrier function. SCFAs produced by intestinal flora fermentation provide energy for intestinal epithelial cells, promote their proliferation, maintain intestinal barrier function, maintain the stability of the intestinal environment, improve immune tolerance and help to prevent a chronic intestinal inflammatory response to microorganisms and their products [47].

Another confirmed major signaling mechanism of SCFAs is preventing the activation of G-protein-coupled receptors (GPCRs). GPR41, GPR43 and GPR109A receptors were identified as SCFA receptors. Acetic acid, propionic acid and butyric acid activate GPR41 and GPR43 receptors, commonly expressed in secretory cells, adipocytes and macrophages, and butyric acid activates GPR109A on the surface of colon cells, adipocytes and stem cells [48]. Once these receptors recognize SCFAs, they immediately activate the signal transduction pathway of the immune response and promote the differentiation of CD4 + T cells into Treg cells and Th cells. This is a kind of T lymphocyte with CD4 + T molecules on the surface. These CD4+ T cells, also known as the “helpers” of the immune system, direct the body to fight pathogenic microorganisms. The number of CD4 + T cells directly reflects the immune function of the human body [49]. Signal transduction acts on DCs to improve their tolerance so as to enhance the immune response in vivo. SCFAs have also been shown to enhance the function of Th1 and Treg cells [50].

SCFAs are famous histone deacetylase (HDAC) inhibitors, which stimulate histone acetyltransferase activity, inhibit HDAC activation and stabilize hypoxia-inducible factor (HIF). HDAC is an enzyme that separates acetyl and acetyllysine amino acids from histones and non-histones [51]. It is used to change nucleosome conformation and regulate gene expression. Its mechanism is to deacetylate histone, tightly bind it to negatively charged DNA and inhibit gene transcription. Therefore, SCFAs stimulate monocytes and neutrophils, resulting in the inactivation of the NF-kB signaling pathway [52]. NF-kB is involved in inflammatory and immune responses. NF-kB overactivation causes adverse reactions, such as inflammation. The inactivation of the NF-kB pathway finally reduces IL-2, IL-6 and tumor necrosis factor-α (TNF-α) expression, which are proinflammatory factors. It provides a new method for the treatment of the inflammatory response, as it makes the intestinal homeostasis tend to balance and restores the normality of the original intestinal immune system.

**Figure 2:**
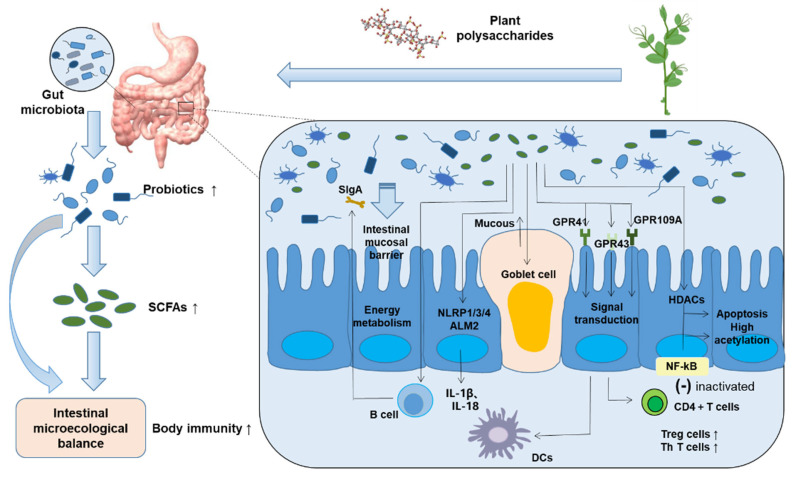
Probiotics and SCFAs participate in various immune-related physiological and biochemical reactions.

As shown in Figure 2, when the human body ingests indigestible herbal polysaccharides, intestinal probiotics will use dietary polysaccharides to produce important metabolic compounds, SCFAs. Probiotics induce goblet cells to secrete mucin and enhance intestinal barrier function. In addition, SCFAs (1) make B cells produce SIgA and strengthen resistance [53]; (2) activate the inflammasome to produce cytokines such as IL-18 and reduce the level of proinflammatory factors in vivo [54]; (3) activate the GPR signaling pathway, promote immune cell differentiation and participate in a variety of immune activities; and (4) inhibit the transmission of the NF-kB signaling pathway and participate in the responses of cells to external stimuli such as cytokines, viruses or bacterial antigens [55].

## 4. Gut Flora Regulates Host Immune System

When external pathogens, such as viruses, attempt to invade the host, they must pass through the intestinal mucosa. Various microbiota on intestinal mucosa provide an effective barrier against pathogen invasion in healthy humans [56]. For example, lactic acid bacteria secrete bacteriocins, and Bifidobacteria secrete acetic acid. Probiotics adhere to the mucosal surface, reducing the ability of pathogens and their toxins to adhere to the intestine. Most probiotics stimulate B cells to produce secretory immunoglobulin A (SIgA). SlgA binds to foreign body antibodies and prevents them from entering epithelial cells. At the same time, probiotics in the intestine bind to invading viruses and prevent the interaction of receptors on pathogen and host cells [57]. In children with rotavirus-induced viral gastroenteritis, the frequency and duration of diarrhea in children are significantly reduced because probiotics stimulate a rotavirus-specific SIgA antibody response. An experiment studying recurrent respiratory tract infections (RRTI), common diseases in preschool children, found that the number of Bifidobacteria and Lactobacilli was significantly lower in the patient group compared to the healthy group. Children with RRTI are accompanied by intestinal flora disturbance, and oral probiotics can effectively improve the intestinal microecological balance and reduce the incidence of RRTI [58]. In addition, probiotics can be used as drugs to limit respiratory viral infections by enhancing mucosal immunity [59].

Intestinal microorganisms secrete antimicrobial peptides and compete for nutrition and habitats to proliferate so as to form a dynamic balance in the body. Intestinal flora-derived signals regulate the proinflammatory response of immune cells, thus affecting the susceptibility to various diseases. Intestinal immune homeostasis is coordinated by regulating the regulatory balance of the proinflammatory response, such as Th1 T cells and Treg cells. There are three main ways for DCs to recognize bacteria: 1. DCs are transferred to DCs in Peyer’s patches (PPs) by phagocytizing micro gold cells on the lumen surface of PPs. As a special epithelial cell, M cells regulate the absorption of DCs by bacteria and intestinal lymphoid tissue; 2. DCs distributed in intestinal epithelial lamina propria (LP) extend synapses from luminal epithelial cells and make contact with bacteria; 3. bacterial metabolites directly enter intestinal mucosal LP through a paracellular pathway and then react with DCs. Different TLRs recognize different bacteria and metabolites. TLRs on the surface of DCs recognize a variety of pathogen-related molecular patterns (PAMPs). In case of pathogen invasion, TLRs produce a variety of signal transductions and gene expression through the recognition of PAMPs and finally produce the following effects: the expression and secretion of a variety of proinflammatory cytokines, such as tumor necrosis factor-α (TNF-α), IL-12 and so on [60]. Multi-omics integration revealed significant associations between bacterial species and metabolic phenotypes, highlighting a key role for the microbiome in modulating human immunity. Another experiment showed that mononuclear phagocytes in the non-mucosal lymphoid organs of mice living in a sterile environment were unable to induce the expression of a set of genes for the inflammatory response, including the encoding of various types of interferons, thus making NK cell initiation and antiviral effects ineffective. However, the mice had a normal immune effect when the gut microbiota of both sterile and normal mice was given in order to normalize and stabilize the number and variety of the mice’s gut microbiota [61].

## 5. Dietary Intervention to Maintain Intestinal Microecosystem Stability and Prevent COVID-19

Altering the gut–lung axis through the gut microbiome may potentially protect humans from respiratory tract infections, and clinical trials of probiotics have shown promise in healthy adults and children. In one study, gut microbial diversity remained stable in participants treated with probiotics. The data from this experiment provide support for further trials to assess the potential role of probiotics in the prevention of COVID-19 [62]. A considerable number of patients with COVID-19 suffer from severe intestinal diseases. This is because intestinal epithelial cells, especially those in the small intestine, also have ACE2 receptors that bind to SARS-CoV-2. The virus invades the intestine, causing disturbances in the intestinal microecological environment. Improving intestinal health through external intake of dietary polysaccharides may provide another unique perspective for the treatment or improvement of COVID-19. Intestinal microbes adapt to the body’s immune system, and the dynamic and stable intestinal microecological environment is interconnected with the powerful immune system. The imbalance of intestinal flora will directly lead to a decline in immunity. Adverse external factors, such as life and rest disorder, unbalanced diet, and abuse of antibiotics and other drugs, will lead to intestinal flora disorder [63], lead to decreased immunity and make the host more vulnerable to pathogens in the environment. It is fortunate that there are practical methods to maintain the stability of intestinal microecology, such as fecal microbiome transplantation (FMT), intake of probiotics or synbiotics, and dietary intervention with polysaccharide-rich foods [64]. Clinically, prebiotics, probiotics or synbiotics have been used to treat chronic kidney disease (CKD), which is essentially to improve intestinal flora imbalance and/or increase intestinal barrier permeability [65]. Therefore, regulating intestinal flora through a long-term individualized diet is a feasible strategy to improve body immunity, and polysaccharides are the key to regulating the microecological balance.

Vegetables and fruits are good sources of prebiotics, including spinach, onion, strawberry, banana, etc. In addition, some scholars found that the health symptoms of mice on a diet supplemented with whole grains were more pronounced [66]. Combined with human intervention studies, dietary fiber and whole-grain intake increase the diversity of intestinal bacteria [67]. In Western society, it is generally believed that low fiber intake will lead to increased consumption of gastrointestinal microbiota and increase the risk of chronic non-communicable diseases [68].

In China, it has long been known that plant polysaccharides have satisfactory therapeutic effects. They are widely used in the prevention and treatment of various diseases. For example, the flower head of chrysanthemum is the most representative flower derivative, which is mainly used to treat respiratory and cardiovascular diseases. It has significant antibacterial, anti-inflammatory, anticancer and neuroprotective activities [69]. This is due to chrysanthemum polysaccharide. Chinese people like to put chrysanthemum heads and air-dried medlar in hot water to drink later. Astragalus polysaccharide (APS) can directly kill tumor cells and improve immune function and has attracted more and more attention in the field of anticancer treatment. APS significantly promoted the proliferation of mouse spleen lymphocytes and improved the phagocytosis of peritoneal macrophages [70]. Astragalus membranaceus is a rhizome plant. Chinese people often add dried and clean Astragalus to their daily diet, such as Astragalus in soup and porridge. In previous studies, Rehmannia glutinosa polysaccharide (RGP) was found to induce DC maturation, so RGP can be used as an immunostimulatory molecule [71]. In order to make RGP more convenient for human consumption, Chinese people often make Rehmannia glutinosa into pills. In a characterization experiment on rats with yam polysaccharide, the results showed that yam polysaccharide is a good carbon and energy source, which can improve bacterial colony diversity and regulate the production of SCFAs in the rat hindgut [72]. The method of making it edible is also relatively simple. After clearing the topsoil, it is very healthy when cooked with high-temperature steam using the unique cooking methods of China. It was reported that when yam polysaccharide was ingested by mice, it promoted the number of beneficial bacteria and inhibited the growth of pathogens. In addition, from studies on the impact of yam polysaccharide on fecal microorganisms, it was found that yam polysaccharide improved the richness and diversity of colonies through colony technology. Meanwhile, compared with the model group, yam polysaccharide reduced the number of Enterococcus and Clostridium perfringens but increased the number of *Bifidobacteria* and *Lactobacillus* [73]. Coptis polysaccharide can not only promote the growth of PPs but also promote the secretion of IL-17 and TGF-α. It can also be absorbed and utilized by intestinal flora so as to regulate the diversity of intestinal flora. This indicates that coptis chinensis polysaccharide can effectively, dynamically and dose-dependently regulate the intestinal flora [74]. These plant polysaccharides may contribute to the treatment of COVID-19 in the future. Ultimately, when using polysaccharides from plants, attention should be paid to scientifically and reasonably control and arrange the types and doses of plant polysaccharides.

## 6. Conclusions

Plant polysaccharides and their metabolites, SCFAs, can repair the damaged intestinal barrier, reduce the level of inflammation in the body, enhance the body’s immunity and resist viral infection. Furthermore, no disease should be treated in isolation, as organs interact with each other through various networks of connections. Understanding the mechanisms of crosstalk between gut–lung defenses and the role of the gut microbiota in regulating and maintaining immune system homeostasis is a promising area of research in the fight against COVID-19. Although the immunomodulatory effects of plant polysaccharides on gut microbiota have been revealed, the mechanism of SARS-CoV-2 viral infection remains unclear. The use of plant polysaccharides for immune regulation of gut flora, or as an important auxiliary means, may have potential in the prevention and treatment of COVID-19.

## Abbreviations

COVID-19: coronavirus disease 2019; MERS: Middle East respiratory syndrome; WHO: World Health Organization; SCFAs: short-chain fatty acids; BT: bacterial translocation; GALTs: gut-associated lymphoid tissues; APCs: antigen-presenting cells; PPs: Peyer’s patches; DCs: dendritic cells; LP: lamina propria; cDC: traditional DC; TLRs: toll-like receptors; PAMPs: pathogen-associated molecular patterns; TNF-α: tumor necrosis factor; IL-12: interleukin-12; ARDS: acute respiratory distress syndrome; SIgA: secretory immunoglobulin A; GIT: gastrointestinal tract; TMAO: trimethylamine oxide; Treg: regulatory T cells; Th1: helper 1 T cells; Th2: helper 2 T cells; immunoglobulin A: IgA; programmed cell death-1: PD-1; T follicular helper cells: T (FH); CLP: glucagon-like peptide; HDAC: histone deacetylase; HIF: hypoxia-inducible factor; GPCR: G-protein-coupled receptor; FMT: fecal microbiome transplantation; CKD: chronic kidney disease; APS: astragalus polysaccharide; RGP: rehmannia glutinosa polysaccharide.

## References

[1] Huang C., Wang Y., Li X., Ren L., Zhao J., Hu Y., Zhang L., Fan G., Xu J., Gu X. (2020). Clinical features of patients infected with 2019 novel coronavirus in Wuhan, China. Lancet.

[2] Hamid S., Mir M.Y., Rohela G.K. (2020). Novel coronavirus disease (COVID-19): A pandemic (epidemiology, pathogenesis and potential therapeutics). New Microbes New Infect..

[3] Russell C.D., Millar J.E., Baillie J.K. (2020). Clinical evidence does not support corticosteroid treatment for 2019—nCoV lung injury. Lancet.

[4] Dhar D., Mohanty A. (2020). Gut microbiota and COVID-19—possible link and implications. Virus Res..

[5] Chan J.F., Yuan S., Kok K.H., To K.K., Chu H., Yang J., Xing F., Liu J., Yip C.C., Poon R.W. (2020). A familial cluster of pneumonia associated with the 2019 novel coronavirus indicating person-to-person transmission: A study of a family cluster. Lancet.

[6] Chilamakuri R., Agarwal S. (2021). COVID-19: Characteristics and Therapeutics. Cells.

[7] Lankelma J.M., Birnie E., Weehuizen T.A.F., Scicluna B.P., Belzer C., Houtkooper R.H., Roelofs J.J.T.H., de Vos A.F., van der Poll T., Budding A.E. (2020). The gut microbiota as a modulator of innate immunity during melioidosis. PLoS Neglect. Trop Dis..

[8] Turner J.R. (2009). Intestinal mucosal barrier function in health and disease. Nat. Rev. Immunol..

[9] Riksen N.P., Netea M.G. (2021). Immunometabolic control of trained immunity. Mol. Aspects Med..

[10] Mörkl S., Butler M.I., Holl A., Cryan J.F., Dinan T.G. (2020). Probiotics and the microbiota-gut-brain axis: Focus on Psychiatry. Curr. Nutr. Rep..

[11] Mujagic Z., de Vos P., Boekschoten M.V., Govers C., Pieters H.H., de Wit N.J., Bron P.A., Masclee A.A., Troost F.J. (2017). The effects of Lactobacillus plantarum on small intestinal barrier function and mucosal gene transcription; A randomized double-blind placebo controlled trial. Sci. Rep..

[12] Del Piano M., Carmagnola S., Anderloni A., Andorno S., Ballarè M., Balzarini M., Montino F., Orsello M., Pagliarulo M., Sartori M. (2010). The use of probiotics in healthy volunteers with evacuation disorders and hard stools: A double-blind, randomized, placebo-controlled study. J. Clin. Gastroenterol..

[13] Klaenhammer T.R., Kleerebezem M., Kopp M.V., Rescigno M. (2012). The impact of probiotics and prebiotics on the immune system. Nat. Rev. Immunol..

[14] Żółkiewicz J., Marzec A., Ruszczyński M., Feleszko W. (2020). Postbiotics—A Step Beyond Pre- and Probiotics. Nutrients.

[15] Gibson G.R., Hutkins R., Sanders M.E., Prescott S.L., Reimer R.A., Salminen S.J., Scott K., Stanton C., Swanson K.S., Cani P.D. (2017). Expert consensus document: The International Scientific Association for Probiotics and Prebiotics (ISAPP) consensus statement on the definition and scope of prebiotics. Nat. Rev. Gastroenterol. Hepatol..

[16] Yin M., Zhang Y., Li H. (2019). Advances in research on immunoregulation of macrophages by plant polysaccharides. Front. Immunol..

[17] Markowiak P., Śliżewska K. (2017). Effects of Probiotics, Prebiotics, and Synbiotics on Human Health. Nutrients.

[18] Akour A. (2020). Probiotics and COVID-19: Is there any link?. Lett. Appl. Microbiol..

[19] Vergara-Buenaventura A., Castro-Ruiz C. (2020). Use of mouthwashes against COVID-19 in dentistry. Br. J. Oral Maxillofac. Surg..

[20] Yuen K.S., Ye Z.W., Fung S.Y., Chan C.P., Jin D.Y. (2020). SARS-CoV-2 and COVID-19: The most important research questions. Cell Biosci..

[21] Villapol S. (2020). Gastrointestinal symptoms associated with COVID-19: Impact on the gut microbiome. Transl. Res..

[22] Kazmierczak S.K., Vitale E., Makarewicz W. (2020). COVID-gastrointestinal and gut microbiota-related aspects. Eur. Rev. Med. Pharmacol. Sci..

[23] Bozkurt H.S., Quigley E.M. (2020). The probiotic Bifidobacterium in the management of Coronavirus: A theoretical basis. Int. J. Immunopathol. Pharmacol..

[24] Barbosa J.R., de Carvalho R.N. (2021). Polysaccharides obtained from natural edible sources and their role in modulating the immune system: Biologically active potential that can be exploited against COVID-19. Trends Food Sci. Technol..

[25] Song S., Peng H., Wang Q., Liu Z., Dong X., Wen C., Ai C., Zhang Y., Wang Z., Zhu B. (2020). Inhibitory activities of marine sulfated polysaccharides against SARS-CoV-2. Food Funct..

[26] Yim S.K., Kim K., Kim I.H., Chun S.H., Oh T.H., Kim J.U., Kim J.W., Jung W.H., Moon H.S., Ku B.S. (2021). Inhibition of SARS-CoV-2 Virus Entry by the Crude Polysaccharides of Seaweeds and Abalone Viscera In Vitro. Mar. Drugs.

[27] Mohan K., Muralisankar T., Uthayakumar V., Chandirasekar R., Revathi N., Ganesan A., Velmurugan K., Sathishkumar P., Jayakumar R., Seedevi P. (2020). Trends in the extraction, purification, characterisation and biological activities of polysaccharides from tropical and sub-tropical fruits—A comprehensive review. Carbohydr. Polym..

[28] Su Y., Li L. (2020). Structural characterization and antioxidant activity of polysaccharide from four auriculariales. Carbohydr. Polym..

[29] Bezerra I.L., Caillot A.R.C., Palhares L.C.G.F., Santana-Filho A.P., Chavante S.F., Sassaki G.L. (2018). Structural characterization of polysaccharides from Cabernet Franc, Cabernet Sauvignon and Sauvignon Blanc wines: Anti-inflammatory activity in LPS stimulated RAW 264.7 cells. Carbohydr. Polym..

[30] Wang N., Zhang X., Wang S., Guo Q., Li Z., Liu H., Wang C. (2020). Structural characterisation and immunomodulatory activity of polysaccharides from white asparagus skin. Carbohydr. Polym..

[31] Chakka V.P., Zhou T. (2020). Carboxymethylation of polysaccharides: Synthesis and bioactivities. Int. J. Biol. Macromol..

[32] Sanders M.E., Merenstein D.J., Reid G., Gibson G.R., Rastall R.A. (2019). Probiotics and prebiotics in intestinal health and disease: From biology to the clinic. Nat. Rev. Gastroenterol. Hepatol..

[33] Sun Y., Cheng L., Zeng X., Zhang X., Liu Y., Wu Z., Weng P. (2021). The intervention of unique plant polysaccharides-dietary fiber on depression from the gut-brain axis. Int. J. Biol. Macromol..

[34] Holscher H.D. (2017). Dietay fiber and prebiotics and the gastrointestinal microbiota. Gut Microbes.

[35] Tan X., Zhou X., Chen H.G. (2017). Structure-activity relationship of plant polysaccharides. Zhongguo Zhongyao Zazhi.

[36] Song Q., Wang Y., Huang L., Shen M., Yu Y., Yu Q., Chen Y., Xie J. (2021). Review of the relationships among polysaccharides, gut microbiota, and human health. Food Res. Int..

[37] Wang Y.J., Li Q.M., Zha X.Q., Luo J.P. (2022). Dendrobium fimbriatum Hook polysaccharide ameliorates dextran-sodium-sulfate-induced colitis in mice via improving intestinal barrier function, modulating intestinal microbiota, and reducing oxidative stress and inflammatory responses. Food Funct..

[38] Xie X., He Y., Li H., Yu D., Na L., Sun T., Zhang D., Shi X., Xia Y., Jiang T. (2019). Effects of prebiotics on immunologic indicators and intestinal microbiota structure in perioperative colorectal cancer patients. Nutrition.

[39] Vanegas S.M., Meydani M., Barnett J.B., Goldin B., Kane A., Rasmussen H., Brown C., Vangay P., Knights D., Jonnalagadda S. (2017). Substituting whole grains for refined grains in a 6-wk randomized trial has a modest effect on gut microbiota and immune and inflammatory markers of healthy adults. Am. J. Clin. Nutr..

[40] Dongarrà M.L., Rizzello V., Muccio L., Fries W., Cascio A., Bonaccorsi I., Ferlazzo G. (2013). Mucosal immunology and probiotics. Curr. Allergy Asthma Rep..

[41] Maldonado G.C., Cazorla S.I., Lemme D., Vélez E., Perdigón G. (2019). Beneficial effects of probiotic consumption on the immune system. Ann. Nutr. Metab..

[42] Dumas A., Bernard L., Poquet Y., Lugo-Villarino G., Neyrolles O. (2018). The role of the lung microbiota and the gut-lung axis in respiratory infectious diseases. Cell Microbiol..

[43] Karakula-Juchnowicz H., Rog J., Juchnowicz D., Łoniewski I., Skonieczna-Żydecka K., Krukow P., Futyma-Jedrzejewska M., Kaczmarczyk M. (2019). The study evaluating the effect of probiotic supplementation on the mental status, inflammation, and intestinal barrier in major depressive disorder patients using gluten-free or gluten-containing diet (SANGUT study): A 12-week, randomized, double-blind, and placebo-controlled clinical study protocol. Nutr. J..

[44] Cui L., Guan X., Ding W., Luo Y., Wang W., Bu W., Song J., Tan X., Sun E., Ning Q. (2021). Scutellaria baicalensis Georgi polysachharide ameliorates DSS-induced ulcerative colitis by improving intestinal barrier function and modulating gut microbiota. Int. J. Biol. Macromol..

[45] Holota Y., Dovbynchuk T., Kaji I., Vareniuk I., Dzyubenko N., Chervinska T., Zakordonets L., Stetska V., Ostapchenko L., Serhiychuk T. (2019). The long-term consequences of antibiotic therapy: Role of colonic short-chain fatty acids (SCFA) system and intestinal barrier integrity. PLoS ONE.

[46] Shinde T., Perera A.P., Vemuri R., Gondalia S.V., Beale D.J., Karpe A.V., Shastri S., Basheer W., Southam B., Eri R. (2020). Synbiotic supplementation with prebiotic green banana resistant starch and probiotic bacillus coagulans spores ameliorates gut inflammation in mouse model of inflammatory bowel diseases. Eur. J. Nutr..

[47] Parada Venegas D., De la Fuente M.K., Landskron G., González M.J., Quera R., Dijkstra G., Harmsen H.J.M., Faber K.N., Hermoso M.A. (2019). Short chain fatty (SCFAs)-mediated gut epithelial and immune regulatiom and its relevance for inflammatory bowel diseases. Front. Immunol..

[48] Kim M.H., Kang S.G., Park J.H., Yanagisawa M., Kim C.H. (2013). Short-chain fatty acids activate GPR41 and GPR43 on intestinal epithelial cells to promote inflammatory responses in mices. Gastroenterology.

[49] Tan J., McKenzie C., Potamitis M., Thorburn A.N., Mackay C.R., Macia L. (2014). The role of short-chain fatty acids in health and disease. Adv. Immunol..

[50] Smelt M.J., de Haan B.J., Bron P.A., van Swam I., Meijerink M., Wells J.M., Faas M.M., de Vos P. (2013). Probiotics can generate foxp3 T-cell responses in the small intestine and simultaneously inducing CD4 and CD8 T cell activation in the large intestine. PLoS ONE.

[51] Ratajczak W., Rył A., Mizerski A., Walczakiewicz K., Sipak O., Laszczyńska M. (2019). Immunomodulatory potential of gut microbiome-derived short chain fatty acids (SCFAs). Acta Biochim. Pol..

[52] Kumar J., Rani K., Datt C. (2020). Molecular link between dietary fibre, gut microbiota and health. Mol. Biol. Rep..

[53] Mio K., Otake N., Nakashima S., Matsuoka T., Aoe S. (2021). Ingestion of High β-Glucan Barley Flour Enhances the Intestinal Immune System of Diet-Induced Obese Mice by Prebiotic Effects. Nutrients.

[54] Liu L., Li Q., Yang Y., Guo A. (2021). Biological Function of Short-Chain Fatty Acids and Its Regulation on Intestinal Health of Poultry. Front. Vet. Sci..

[55] Huang W., Man Y., Gao C., Zhou L., Gu J., Xu H., Wan Q., Long Y., Chai L., Xu Y. (2020). Short-Chain Fatty Acids Ameliorate Diabetic Nephropathy via GPR43-Mediated Inhibition of Oxidative Stress and NF-κB Signaling. Oxid Med. Cell Longevity.

[56] Marsland B.J., Trompette A., Gollwitzer E.S. (2015). The gut-lung axis in respiratory disease. Ann. Am. Thorac Soc..

[57] Kusumo P.D., Bela B., Wibowo H., Munasir Z., Surono I.S. (2019). Lactobacillus plantarum IS-10506 supplementation increase faecal SIgA and immune response in children younger than two years. Benefic Microbes.

[58] Li K.L., Wang B.Z., Li Z.P., Li Y.L., Liang J.J. (2019). Alterations of intestinal flora and the effects of probiotics in children with recurrent respiratory tract infection. World J. Pediatr..

[59] Enaud R., Prevel R., Ciarlo E., Beaufils F., Wieërs G., Guery B., Delhaes L. (2020). The gut-lung axis in health and respiratory diseases: A place for inter-organ and inter-kingdom crosstalks. Front. Cell Infect. Microbiol..

[60] Chang C.S., Kao C.Y. (2019). Current understanding of the gut microbiota shaping mechanisms. J. Biomed. Sci..

[61] Ganal S.C., Sanos S.L., Kallfass C., Oberle K., Johner C., Kirschning C., Lienenklaus S., Weiss S., Staeheli P., Aichele P. (2012). Priming of natural killer cells by nonmucosal mononuclear phagocytes requires instructive signals from commensal microbiota. Immunity.

[62] Mullish B.H., Marchesi J.R., McDonald J.A.K., Pass D.A., Masetti G., Michael D.R., Plummer S., Jack A.A., Davies T.S., Hughes T.R. (2021). Probiotics reduce self-reported symptoms of upper respiratory tract infection in overweight and obese adults: Should we be considering probiotics during viral pandemics?. Gut Microbes.

[63] Ray D., Kidane D. (2016). Gut microbiota imbalance and base excision repair dynamics in colon cancer. J. Cancer.

[64] Fong W., Li Q., Yu J. (2020). Gut microbiota modulation: A novel strategy for prevention and treatment of colorectal cancer. Oncogene.

[65] Guldris S.C., Parra E.G., Amenós A.C. (2017). Gut microbiota in chronic kidney disease. J. Bras. Nefrol..

[66] Sang S., Idehen E., Zhao Y., Chu Y. (2020). Emerging science on whole grain intake and inflammation. Nutr. Rev..

[67] Tap J., Furet J.P., Bensaada M., Philippe C., Roth H., Rabot S., Lakhdari O., Lombard V., Henrissat B., Corthier G. (2015). Gut microbiota richness promotes its stability upon increased dietary fibre intake in healthy adults. Environ. Microbiol..

[68] Deehan E.C., Walter J. (2016). The fiber gap and the disappearing gut microbiome: Implications for human nutrition. Trends Endocrinol. Metab..

[69] Yuan H., Jiang S., Liu Y., Daniyal M., Jian Y., Peng C., Shen J., Liu S., Wang W. (2020). The flower head of Chrysanthemum morifolium Ramat. (Juhua): A paradigm of flowers serving as Chinese dietary herbal medicine. J. Ethnopharmacol..

[70] Li W., Hu X., Wang S., Jiao Z., Sun T., Liu T., Song K. (2019). Characterization and anti-tumor bioactivity of astragalus polysaccharides by immunomodulation. Int. J. Biol. Macromol..

[71] Wang H., Lian P., Niu X., Zhao L., Mu X., Feng B., Li J., Liang Z., Qiao J. (2018). TLR4 deficiency reduces pulmonary resistance to streptococcus pneumoniae in gut microbiota-disrupted mice. PLoS ONE.

[72] Kong X.F., Zhang Y.Z., Wu X., Yin Y.L., Tan Z.L., Feng Y., Yan F.Y., Bo M.J., Huang R.L., Li T.J. (2009). Fermentation characterization of chinese yam polysaccharide and its effects on the gut microbiota of rats. Int. J. Microbiol..

[73] Wagley S., Bokori-Brown M., Morcrette H., Malaspina A., D’Arcy C., Gnanapavan S. (2018). Evidence of clostridium perfringens epsilon toxin associated with multiple sclerosis. Mult. Scler..

[74] Chen Q., Ren R., Zhang Q., Wu J., Zhang Y., Xue M., Yin D., Yang Y. (2021). Coptis chinensis Franch polysaccharides provide a dynamically regulation on intestinal microenvironment, based on the intestinal flora and mucosal immunity. J. Ethnopharmacol..

